# Molecular docking based design of Dengue NS5 methyltransferase inhibitors

**DOI:** 10.6026/97320630015394

**Published:** 2019-05-30

**Authors:** Mohd Adnan Kausar, Abrar Ali, Samir Qiblawi, SMA Shahid, Mohammad Asrar Izhari, Anamika saral

**Affiliations:** 1 College of Medicine, University of Hail, Hail, KSA; 2 Department of Laboratory Medicine College of Applied Medical Science, Al-Baha University, Saudi Arabia; 3 School of studies in Biotechnology, Jiwaji University, Gwalior, Madhya Pradesh, India

**Keywords:** Dengue, methyltransferase, molecular docking, virtual Screening, IBS

## Abstract

Dengue is a viral infection caused by RNA infection of the family Flaviviridae and spread by the Aedes mosquitoes. Dengue NS5 methyltransferase is a known drug target for the disease. Therefore, it is of interest to design potential inhibitors for the target using molecular docking analysis. Our analysis shows the binding of compounds STOCK1N-98943, STOCK1N-98872, STOCK1N-98956, STOCK1N-98865, and STOCK1N-98950 with the protein drug target with optimal binding features for further in vitro and in vivo evaluations.

## Background

The dengue infection is caused by Flavivirus of the family Flaviviridae and it is an arthropod-borne diseases that consolidates four particular serotypes (DEN-1, DEN-2, DEN-3, and DEN-4) 1, 2. The World Health Organization (WHO) considers dengue as a significant overall general prosperity challenge in the tropic and subtropics nations. Dengue is seen as a result of an unnatural climate change, unconstrained urbanization, inefficient mosquito control, and non appearance of social insurance facilities. 3-5. Two and a half billion individuals live in dengue-endemic regions 6 and about 400 million contaminations occurring every year, with a death rate out-performing 5-20% in some areas 7. Dengue disease is seen in excess of 100 nations including Europe and the United States (USA) 2. The essential and non-fundamental proteins of DENV have been focal point of an antiviral structure. The DENV non-assistant proteins (NS1, NS2A, NS2B, NS3, NS4A, NS4B and NS5) have role in replication and virion assembly 8. Screening of compounds as antiviral drugs is gaining momentum in recent years. Peptide-based drugs with higher bioactivity and low lethality has also been considered 9. Compounds with anti-viral effects for the avian flu infection subtype H9N2 10 and subtype H5N1 11 are also known. Therefore, it is of interest in this context to screen for compounds against the Dengue NS5 methyltransferase as potential inhibitors for consideration as anti-viral drugs.

## Methodology

### Domain organization:

The methyltransferase domain contains 262 aminoacid residues. The FASTA format sequence was further analyzed using Pfam, Prosite, SMART, PANTHER and Inter ProScan 12-14.

### Three-dimensional structure prediction by I-TASSER:

The sequence of 262 aminoacid residues of methyltransferase domain (MTD) was retrieved from the Swiss Prot database in FASTA format. The three-dimensional model was generated using the I-TASSER server by multiple threading alignments and iterative structural assembly simulation 15.

### Validation of the predicted model:

The Ramachandaran plot further validated the conformation of the best model predicted by the I-TASSER. The model quality was calculated by analyzing the phi (Φ) and psi (Ψ) torsion angles using the PROCHECK server. The Ramachandaran plot obtained from PROCHECK help select a good quality model with over 90% residues in the favoured region 16. Prediction of the active site of the methyltransferase domain: The active sites in MTD were identified using the computed atlas of surface topography of proteins (CASTp) server (http://cast.engr.uic.edu). It defines all the possible pockets in the protein structure. It measures the area and volume of each pocket and cavity analytically, both in solvent accessible surface and molecular surface 17-19. Here, we input the target protein for predicting the ligand binding sites, and the CASTp server predicts the amino acids crucial for binding interactions and docking studies.

### Prediction of the active site of the methyltransferase domain:

The active sites in MTD were identified using the computed atlas of surface topography of proteins (CASTp) server (http://cast.engr.uic.edu). It defines all the possible pockets in the protein structure. It measures the area and volume of each pocket and cavity analytically, both in solvent accessible surface and molecular surface 17-19. Here, we input the target protein for predicting the ligand binding sites, and the CASTp server predicts the amino acids crucial for binding interactions and docking studies.

### Selection of ligands and protein target molecule:

**Figure 1:**
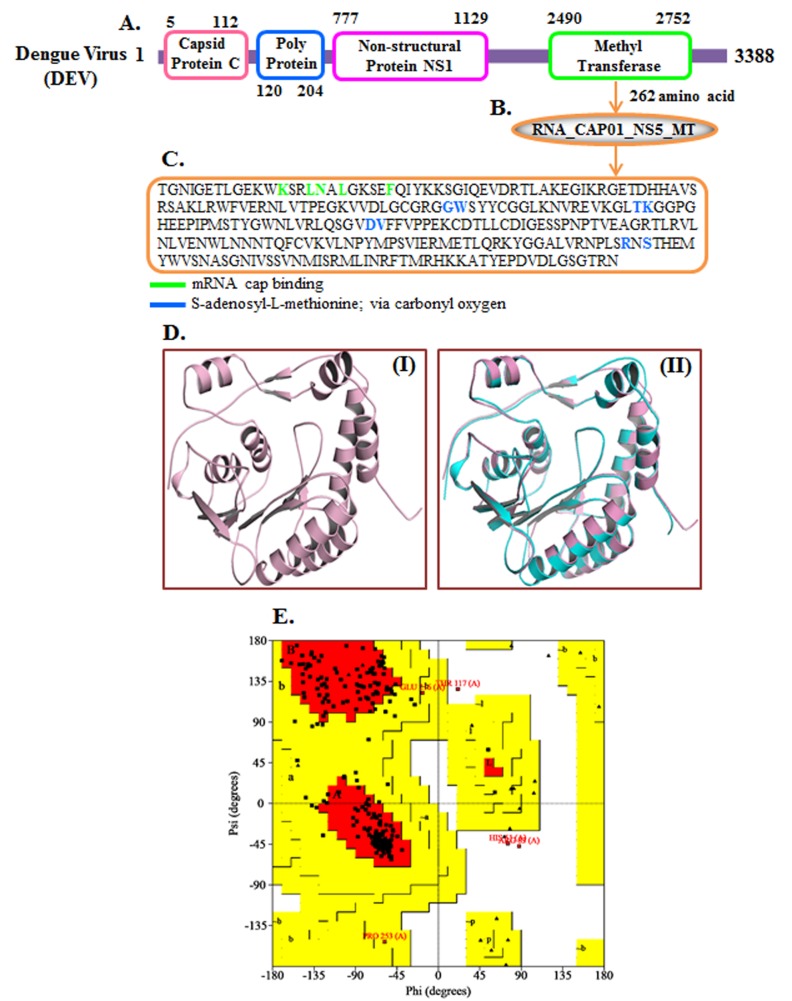
(A) Detailed domain organization of Dengue Virus (DEV). (B) Methyltransferase (MTs) domain contains 262 aminoacid. (C) Amino acid sequence of methyltransferase (MTs) domain essential amino acid is shown in different color (D) (I) three-dimensional structure of MTD protein predicted by I-TASSER. (II) Alignment of query protein (Pink) with structural analog (Cyan) 2px5A in PDB library. (E) Validation of top score model of I-TASSER by PROCHECK Ramachandaran plot of MTD.

The schematic diagram of the workflow design is demonstrated in Figure 1. Ligand molecules were selected from the IBS natural compound library (InterBioScreen Ltd). Ligands were prepared using the LigPrep module of the Maestro 10.5 application. LigPrep performs many corrections on the ligands. These include the addition of hydrogens, 2D-3D conversion, corrected bond lengths and bond angles, low-energy structure and ring conformation. Allatom force field charges and atom types were assigned by the optimized potential for fluid simulations (OPLS_2005) force field 20-23. One conformation for each ligand was thus generated for docking. The structural model of the NS5 methyltransferase generated by ITSER was used in this study. The Maestro 10.5 protein preparation module was used where changes such as the addition of hydrogen atoms, assigning bond orders, creation of zero-order bonds to metal, creation of disulfide bonds, fixing of the charges and orientation of groups were incorporated into the structure.

### Molecular docking:

Molecular docking studies using the selected ligand molecules were completed using Maestro 10.5 molecular docking suite 13, 18, 24-26. Each of these compounds was docked into target protein accordingly with positions, orientations, and conformations of the ligand in the receptor binding site, and the docking structure is possessing the lowest energy was preferred. In the present study, we screened approximately 50,000 natural compounds from the IBS against NS5 methyltransferase. IBS natural compounds docked with every selected protein molecules by using HTVS. To provide a better correlation between right poses and functional scores, GLIDE-XP mode was subsequently used on the selected conformations in HTVS mode. From Gscore, we select 20 compounds for GLIDEXP molecular docking. After the completion of ligands and proteins preparation, a receptor grid file was generated. For running the grid generation module, we have scaled van der Waal (vdW) radii of receptor atoms by 1.00 Å as the default setting of Maestro 10.5. The active site of the receptor maintain a precise scoring function with thermodynamically most favourable energy and is calculated on a grid by various sets of fields. After the formation of the receptor grid file, flexible ligands with rigidreceptor- based molecular docking were performed. The best-fit compounds have been chosen for each target by optimal energy value and types of interactions.

### Absorption, distribution, metabolism, excretion, and toxicity(ADME/T) properties ponders:

Most of the medication applicants don't prevail in clinical preliminaries because of poor toxicology evaluations (ADME/T). In this way, ADME/T properties of best-docked mixes were selected utilizing QikProp utilization of Maestro 10.5 (auxiliary, physicochemical, biochemical, pharmacokinetics, and lethality properties). It predicts inborn properties of the atoms (medicate like properties, for example, octanol/water segment, log BB, by an overall CNS activity, register IC50 for Herg K+ channel blockage, Caco-2, MDCK cell porous features and logKhsa for human serum albumin binding 27, 28.

## Results and discussion:

### Sequence analysis and domain association:

We have analyzed the amino acid sequence of the methyltransferase domain (262 aminoacid) by using different available tools. Figure 1A shows the Dengue virus (DEV) NS5 methyltransferase domain architecture. The InterProScan results show that methyltransferase (MTs) constitute an important class of enzymes present in every life form (Figure 1B). They transfer a methyl group most frequently from S-adenosyl L-methionine (SAM or AdoMet) to a nucleophilic acceptor such as nitrogen, oxygen, sulfur or carbon leading to S-adenosyl-L-homocysteine (AdoHcy) and a methylated molecule. The crucial amino acids for binding are shown using different colours (Figure 1C).

### Three dimensional structure prediction:

The predicted model of MTD protein and its three-dimensional coordinate file in PDB format were successfully obtained from ITASSER. The results obtained from the server includes predicted secondary structure with a confidence score (range 0 to 9), predicted solvent accessibility, five predicted structures with Cscore, top ten templates from PDB used in alignment, high ten PDB structural analogs, functional analogs protein, and binding site residues. Model MTD (Figure 1 D) was selected as the bestpredicted model with C-score 0.99, TM-score 0.85 ± 0.08, and RMSD 2.8 ± 2.1 Å. C-score with higher value reflects a model of better quality. Top ten threading templates for query protein sequence MTD were identified by LOMETS meta-server (Table 1). Normalized Z-score generally estimates the threading alignment. However, a normalized Z-score >1 value reflects a confident, but in case of small alignment of the large query sequence, it does not give a significant indication of modelling accuracy. The percentage sequence identity in the threading aligned region (Iden1) and in the whole chain (Iden2) considered for the excellent homology. The structural alignment program, TM-align, identified 2px5A in PDB library as best structural analog of the top scoring model of ITASSER with the TM-score of 0.792,0.979 and 0.979respectively (Table 2).

### Assessment of predicted model:

The Ramachandaran plots of the best-predicted model were obtained from PROCHECK servers which showed the reliability of the model. The PROCHECK Ramachandaran plot showed 98.2% residues in most favored regions and 0.5% residues in additional allowed regions, i.e., the total of 98.7% residues in allowed regions which indicates a good quality model of MTD (Figure 1 E).

### Screening of targeted compounds against MTD protein:

**Figure 2:**
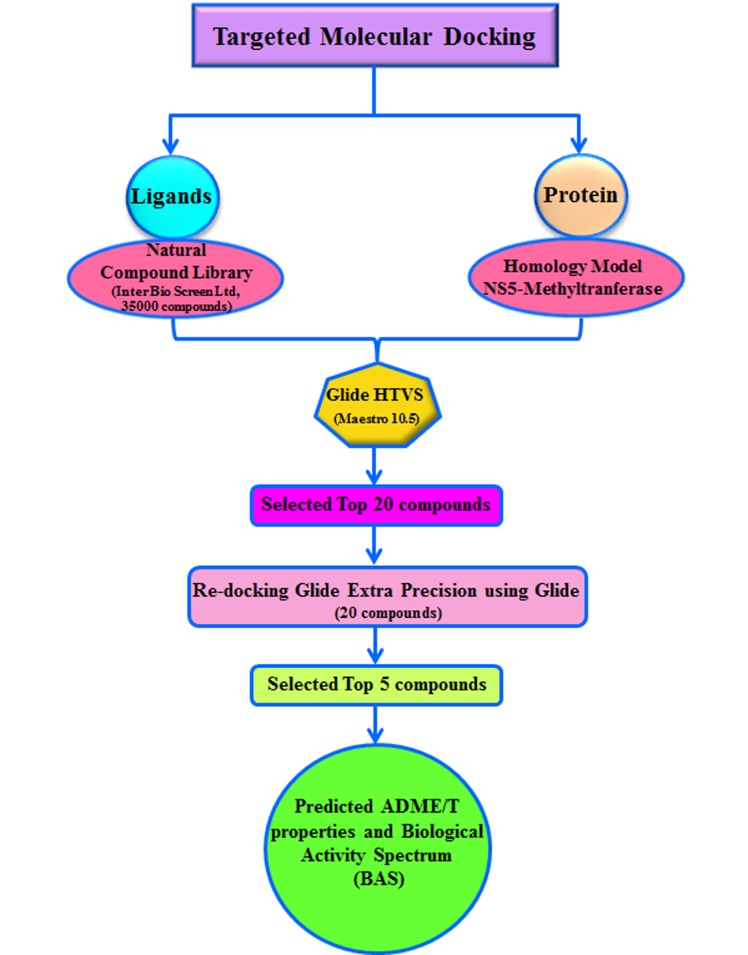
Workflow of screening of targeted compounds against MTD protein.

In the 21st century has the disadvantage that it is challenging to get new compounds into the clinic. The target compounds were identified after virtual screening (VS). This is a reliable, inexpensive method for identifying leads by MTD. Figure 2 clearly reveals that complete flow chart of compound screening.

### Dynamic site:

**Figure 3:**
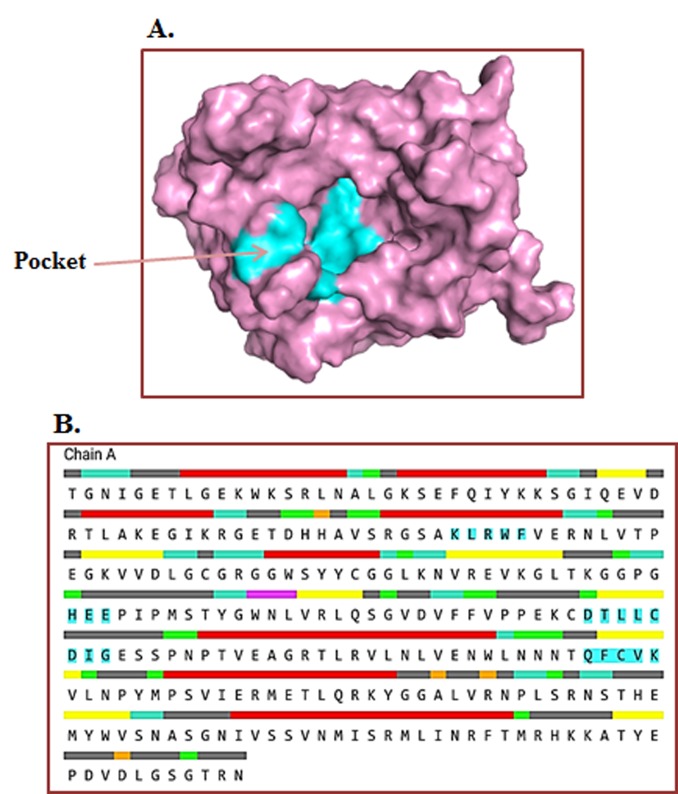
Binding pocket identification by CASTp server: A) Shows the binding sites of MTD protein, (B) Cyan color boxes highlight the amino acid residues present in the binding site

The protuberant binding site of MTD was calculated using the CASTp server with ideal parameters (Figure 3). CASTp evaluation observed the active site amino acids, surface area (473.597) and volume (558.187) of MTD. In MTD protein, all 62 binding pockets were categorized to find the residues with probe 1.4 Å radius. The Cyan color denotes the active site amino acid residues involved in the binding pockets (Figure 3A and Figure 3B).

### Molecular docking analysis:

This study is to identify the potent inhibitor against NS5 methyltransferase using molecular docking. Molecular docking of NS5 methyltransferase against natural compounds has been completed. First, we performed HTVS of IBS against the NS5 methyltransferase shown (Table 6). Further, 20 best compounds that possess minimal G score were performed using the XP mode of GLIDE. Our result shows that compounds with good dock score for protein NS5 methyltransferase (Table 3). Protein-ligand interactions show that the lipophilic, hydrogen bonding, p-p stacking, and cation-p interactions represent a ruling contribution at the active site. Molecular docking operation distinguishes the first docking free energy value (G score) against these receptor molecules. Molecular docking result of NS5 methyltransferase against IBS natural compounds identified that compounds STOCK1N-98943 (N-(2-(3-((S,E)-14,16-dihydroxy-3-methyl-1,7- dioxo-3,4,5,6,7,8,9,10-octahydro-1H-benzo[c][1] oxacyclotetradecin- 15-yl)-3-(4 methoxy phenyl) propanamido)ethyl) benzo [d]thiazole- 2-carboxamide), STOCK1N-98872, STOCK1N-98956, STOCK1N- 98865, and STOCK1N-98950 showed the best G score -8.24, -7.41, - 6.95, -6.73 and -6.52 kcal/mol, respectively. The molecular docking study illustrates the protein�ligands interactions and to summarize the various bonds such as hydrogen and electrostatic bond.

**Figure 4:**
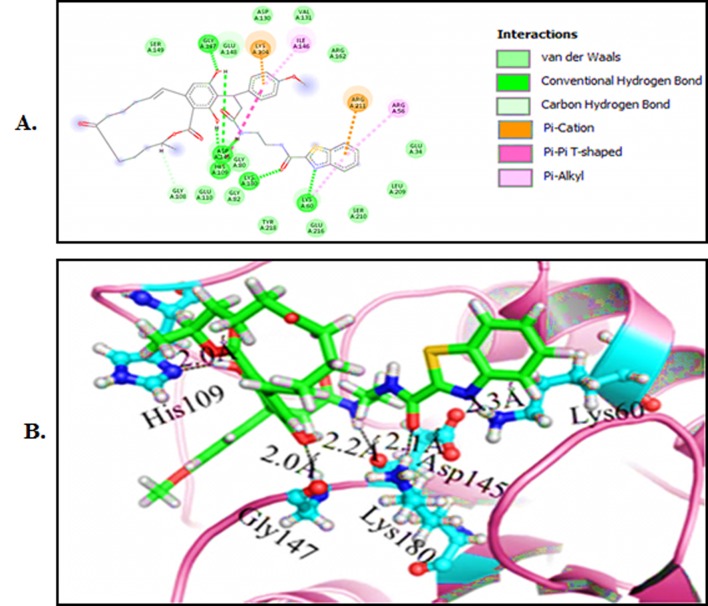
Molecular docking of compounds with MTD: A) (I) 2D schematic diagram showing interactions of compound STOCK1N- 98943. (II) Cartoon view of MTD protein with compound STOCK1N-98943.

STOCK1N-98943 was found to be the most potent and nicely bounded into the active site of NS5 methyltransferase with best G score compared to Chloroquine (Table 3). Compound STOCK1N- 98943 demonstrated six hydrogen bonds with LysA: 60, HisA: 109, AspA: 145, GlyA: 147 and LysA: 180 of NS5 methyltransferase at 2.3 Å, 2.0 Å, 2.1 Å, 2.2 Å, 2.0 Å and 2.2 Å respectively (Figure 4B). The compound STOCK1N-98943 also interacts with the NS5 methyltransferase binding site by interacting with other residues (ArgA: 57, GlyA: 80, LysA: 104, GlyA: 108, and ArgA: 211) as compared to Chloroquine shown in Figure 4A. Chloroquine compound interacts with the NS5 methyltransferase binding site by interacting with residues (AspA:78, CysA:81, TrpA:86, LysA:104, AspA:145 and IleA:146) as shown in Table 1. Molecular docking studies suggested that numerous van der Waals (vdW), covalent, carbon-hydrogen, Pi alkyl, and electrostatic interactions are the critical force for holding of compounds STOCK1N-98943, STOCK1N-98872, STOCK1N-98956, STOCK1N-98865, and STOCK1N-98950 together with the NS5 methyltransferase. Therefore, finally compounds STOCK1N-98943, STOCK1N-98872, STOCK1N-98956, STOCK1N-98865, and STOCK1N-98950 show better binding energy for NS5, and it may be considered as a specific inhibitor of the NS5 methyltransferase.

### ADME properties:

Pharmacokinetic and pharmacodynamics properties of lead compounds were evaluated using the Qikprop application of Maestro 10.5. Compounds STOCK1N-98943, STOCK1N-98872, STOCK1N-98956, STOCK1N-98865, and STOCK1N-98950 yielded the best G score. These compounds have high QPlogPo/w, QPlogHERGK+ channels, QPlogBB, QPlogKP and QPlogKhsa values which satisfy the Lipinski's Rule of Five (Table 4). Moreover, activities such as QPPCaco, QPPMDCK, and percentage oral absorption are satisfactory except STOCK1N-98865. So, structural modification is required for the compound STOCK1N- 98865 to enhance these activities. Polar surface area, high oral bioavailability, H-bond donors, and acceptors are necessary criteria for the development of therapeutic agents. It is reported that compounds with 10 or fewer rotatable bonds and polar surface area equal to or less than 140 Å^2^ have high probability for good oral bioavailability in the rat. These results indicate that these compounds have better permeation rate (Table 4).

### Biological activity predictions:

The selected bioactive constituents were evaluated for possible biological activity using the PASS online server. The biological activity spectrum (BAS) of a compound is known to have pharmacological effects, specific toxicities, and mechanisms of action. Because these probabilities can be calculated independently, the Pa and Pi values vary from 0 to 1, and Pa + Pi < 1. Pa is for the class of active compounds and Pi is for stable compounds 29. PASS prediction results showed that Pa value is higher than Pi value inferring anti-dengue activity of selected compounds (Table 5). It should be noted that all compounds have shown a significant Pa value compared to Pi value with the potential for inhibiting NS5 methyltransferase.

## Conclusion

Dengue has progressed as a common disease affecting about 2.5 billion individuals over 100 countries. Therefore, it is of interest to design potential inhibitor drugs to control and combat Dengue fever. We analyze known structural data using molecular docking and report the binding properties of potential inhibitors to dengue NS5 methyltransferase for further consideration. These inhibitors have optimal binding features with the drug target.

## Conflict of Interest

The authors declare no conflicts of interest.

## Figures and Tables

**Table 1:** Top ten templates used by I-TASSER for threading alignment

Rank	PDB Hit	Iden1	Iden2	Cov.	Z-score
1	4v0qA	0.77	0.76	0.98	3.31
2	4v0rA	0.77	0.76	0.98	4.36
3	4v0qA	0.77	0.76	0.98	3.56
4	4k6m	0.57	0.6	0.97	1.34
5	4k6m	0.53	0.6	0.93	2.07
6	4v0qA	0.77	0.76	0.98	4.08
7	4k6m	0.61	0.6	0.98	3.32
8	4k6mA	0.61	0.6	0.98	5.63
9	4k6mA	0.61	0.6	0.98	3.65
10	4k6mA	0.61	0.6	0.98	4.24

**Table 2:** Top ten structural analogs in PDB identified by TM-align

Rank	PDB Hit	TM-score	RMSDa	IDENa	Cov.
1	2px5A	0.979	0.8	0.625	0.992
2	4v0qA	0.978	0.34	0.771	0.985
3	1l9kA	0.973	0.59	0.973	0.981
4	4k6mA	0.971	0.8	0.606	0.985
5	2oy0B	0.97	0.66	0.636	0.981
6	3evaA	0.968	0.77	0.535	0.981
7	5tfrA	0.967	0.73	0.605	0.981
8	2wa1B	0.966	0.77	0.512	0.981
9	3elyA	0.965	0.82	0.558	0.981
10	3gczA	0.964	0.74	0.556	0.977

**Table 3:** Lowest binding energy for the Ligands-NS5 Methyltranfersaeinteraction, along with scores for various interaction types, as detected by GLIDE

Compounds ID	GScore	Lipophilic E vdw	H-bond	Electro	Protein ligands interaction
STOCK1N-98943	-8.24	-5.24	-1.27	-0.47	ArgA:57, LysA:60, LysA:104, HisA:109, AspA:145, GlyA:147 and ArgA:211
STOCK1N-98872	-7.41	-3.39	-1.92	-0.43	CysA:81, TrpA:86 and ArgA:211
STOCK1N-98956	-6.95	-4.8	-0.7	-0.48	LysA:60 and LysA:104,
STOCK1N-98865	-6.73	-4.9	-0.7	-0.49	TrpA:86, LysA:104 and IleA:146
STOCK1N-98950	-6.52	-3.07	-0.88	-0.46	ArgA:57, AspA:145, GlyA:147 and ArgA:211
Known Inhibitor					
Chloroquine	-5.24	-4.43	-1.97	-0.93	AspA:78, CysA:81, TrpA:86, LysA:104, AspA:145 and IleA:146

**Table 4:** Evaluation of drug-like properties of the lead molecules by Qikprop Maestro 10.5 molecular docking suite

Molecule	QPlogPo/w (-2.0 to 6.5)	Q P log HERG (acceptable range: above -5.0)	QPP Caco (nm/s) <25 - poor >500 - great	Q P log BB (-3 to 1.2)	QPP MDCK (nm/s)	Q Plog Kp (-8.0 to -0.1)
STOCK1N-98943	5.344	-6.281	46.395	-2.886	49.729	-3.431
STOCK1N-98872	3.653	-4.349	49.867	-2.486	35.846	-3.979
STOCK1N-98956	5.335	-5.366	141.335	-1.887	108.881	-0.2687
STOCK1N-98865	2.971	-2.726	23.795	-1.679	18.639	-4.377
STOCK1N-98950	2.358	-6.184	97.867	-1.871	39.817	-3.967

**Table 5:** Biological activity spectrum of compounds (Pa - Active; Pi - Inactive)

Molecule	Pa	Pi	Activity
STOCK1N-98943	0.819	0.029	Antidengue
STOCK1N-98872	0.742	0.022	Antidengue
STOCK1N-98956	0.821	0.008	Antidengue
STOCK1N-98865	0.749	0.016	Antidengue
STOCK1N-98950	0.948	0.029	Antidengue

**Table 6:** Lowest binding energy for the ligands-NS5 Methyltranfersaewith scores for various interaction types, as detected by High throughput virtual screening.

S. No.	Compounds ID	GScore	Lipophilic E vdw	H-bond	Electro
1	STOCK1N-98872	-9.24	-5.24	-1.27	-0.47
2	STOCK1N-98865	-9.11	-3.39	-1.92	-0.43
3	STOCK1N-98871	-8.97	-4.8	-0.7	-0.48
4	STOCK1N-98950	-8.45	-4.9	-0.7	-0.49
5	STOCK1N-98946	-8.08	-3.07	-0.88	-0.46
6	STOCK1N-98870	-7.73	-3.35	-2.81	-1.04
7	STOCK1N-98868	-7.52	-1.55	-3.82	-1.1
8	STOCK1N-98869	-7.36	-3.22	-2.43	-0.95
9	STOCK1N-98956	-7.32	-3.24	-2.37	-1.01
10	STOCK1N-98949	-7.17	-3.67	-1.83	-0.69
11	STOCK1N-98953	-7.03	-4.38	-1.15	-0.41
12	STOCK1N-98944	6.92	-3.12	-1.95	-0.46
13	STOCK1N-98951	6.88	-3.49	-2.07	-0.28
14	STOCK1N-98866	6.76	-1.75	-3.8	-0.81
15	STOCK1N-98943	6.66	-3.86	-1.85	-0.74
16	STOCK1N-98867	6.51	-3.54	-2.58	-1.02
17	STOCK1N-99175	6.43	-2.99	-1.65	-0.58
18	STOCK1N-98865	6.3	-4.04	-1.46	-0.73
19	STOCK1N-98873	6.19	-3.21	-2.81	-0.17
20	STOCK1N-99176	6.11	-3.69	-3.82	-0.8
Known inhibitor					
1	Chloroquine	-5.24	-4.43	-1.97	-0.93
